# Spontaneous Infraslow Fluctuations Modulate Hippocampal EPSP-PS Coupling 

**DOI:** 10.1523/ENEURO.0403-17.2017

**Published:** 2018-01-17

**Authors:** Michael B. Dash, Stephen Ajayi, Lynde Folsom, Paul E. Gold, Donna L. Korol

**Affiliations:** 1 Department of Psychology, Middlebury College, Middlebury, VT 05753; 2 Department of Biology, Syracuse University, Syracuse, NY 13244

**Keywords:** EEG, evoked response, excitability, hippocampus, infraslow, synapse

## Abstract

Extensive trial-to-trial variability is a hallmark of most behavioral, cognitive, and physiological processes. Spontaneous brain activity (SBA), a ubiquitous phenomenon that coordinates levels and patterns of neuronal activity throughout the brain, may contribute to this variability by dynamically altering neuronal excitability. In freely-behaving male rats, we observed extensive variability of the hippocampal evoked response across 28-min recording periods despite maintaining constant stimulation parameters of the medial perforant path. This variability was related to antecedent SBA: increases in low-frequency (0.5–9 Hz) and high-frequency (40.25–100 Hz) band-limited power (BLP) in the 4-s preceding stimulation were associated with decreased slope of the field EPSP (fEPSP) and increased population spike (PS) amplitude. These fluctuations in SBA and evoked response magnitude did not appear stochastic but rather exhibited coordinated activity across infraslow timescales (0.005–0.02 Hz). Specifically, infraslow fluctuations in high- and low-frequency BLP were antiphase with changes in fEPSP slope and in phase with changes in PS amplitude. With these divergent effects on the fEPSP and PS, infraslow SBA ultimately modulates EPSP-PS coupling and thereby enables hippocampal circuitry to generate heterogeneous outputs from identical inputs. Consequently, infraslow SBA appears well suited to dynamically alter sensory selection and information processing and highlights the fundamental role of endogenous neuronal activity for shaping the brain’s response to incoming stimuli.

## Significance Statement

The brain’s response to a given input is variable and enables flexible responses based on past experience and/or current needs. Widespread, dynamic, and ever-present, spontaneous brain activity (SBA) may contribute to response heterogeneity by altering endogenous brain states to modulate subsequent processing of exogenous information. We investigated how spontaneous activity could mechanistically alter information processing by assessing its role in modulating the brain’s response to electrically-evoked activation. We find very slow fluctuations in spontaneous activity affect evoked-response variability by altering the coupling of neuronal input (i.e., depolarization) to subsequent neuronal output (i.e., action potential generation). These results, therefore, characterize a broad mechanism by which SBA can alter sensory and information processing.

## Introduction

Trial-to-trial responses of the brain to the same external stimuli are typically heterogeneous (Fox et al., 2006; Scaglione et al., 2011). This heterogeneity could arise from the stochastic nature of action potential propagation (Faisal and Laughlin, 2007) and synaptic transmission (Destexhe et al., 2001; Fellous et al., 2003) leading to variable neuronal activation following stimulation. Alternatively, the brain’s internal milieu (Fontanini and Katz, 2008), including the levels and patterns of spontaneous brain activity (SBA) during stimulus presentation (Hesselmann et al., 2008), may modulate its response to incoming stimuli. Characterizing how SBA affects the response of the brain to incoming stimuli is critical for understanding information processing and sensory selection within the brain (Sterzer et al., 2009; Jetti et al., 2014).

SBA produces the vast majority of the brain’s energetic demands, highlighting the fundamental importance of task-independent activity for brain function (Raichle, 2011). Electrophysiological measures of SBA demonstrate that the brain produces constitutive activity across wide temporal [from infraslow (<0.1 Hz) to ultra-fast (upwards of 600 Hz)] and spatial scales (Buzsáki and Draguhn, 2004). Activity across these disparate spatial and temporal scales does not occur independently, but rather, exhibits complex spatiotemporal organization that in large part is coordinated by ongoing infraslow activity. Spatially, distinct networks of functionally connected brain regions (Smith et al., 2009), including the default mode network (Raichle, 2015), have been identified through their correlated infraslow fluctuations in resting state fMRI activity. Temporally, the phase of infraslow fluctuations in neocortical EEG activity modulates the amplitude of activity at higher frequencies (>1 Hz; Vanhatalo et al., 2004; Monto et al., 2008). Thus, infraslow activity can exert widespread modulation of the levels and patterns of SBA and consequently may regulate the brain’s response to incoming stimulation.

Perception of external stimuli can fluctuate across infraslow timescales associated with ongoing fluctuations in SBA (for review, see Schroeder and Lakatos, 2009; Sadaghiani and Kleinschmidt, 2013). For example, conscious perception of sporadic somatosensory stimulations exhibits spontaneous infraslow fluctuations that are associated with both the phase of infraslow EEG fluctuations and amplitude of higher frequency (1–40 Hz) EEG power (Monto et al., 2008). The association between performance variability and higher frequency power may indicate that infraslow SBA modulates the brain’s response to incoming stimuli through widespread alterations in neuronal excitability. Consistent with this idea, both spontaneous interictal events recorded from the cortex of epileptic patients (Vanhatalo et al., 2004) and spontaneous hippocampal afterdischarges present in a subset of Wistar rats (Penttonen et al., 1999) fluctuate across infraslow timescales. However, the underlying physiologic mechanisms responsible for infraslow changes in excitability, and for associated perceptual variability, remain unclear. It has been proposed that surface recorded infraslow potentials may arise as a consequence of cortico-thalamic connectivity facilitating long-lasting EPSPs within neocortical dendritic processes (Birbaumer et al., 1990; He and Raichle, 2009). The extent and mechanisms by which infraslow fluctuations in SBA are produced outside of neocortex, and, if present, the effects of these spontaneous fluctuations on the processing of incoming stimuli are poorly characterized.

In the present study, we investigated the relationships between spontaneous hippocampal EEG activity and trial-to-trial variability of evoked responses within the dentate gyrus following electrical stimulation of the medial perforant pathway. This approach allowed us to characterize the effects of SBA on both local processing of incoming stimuli (field EPSP; fEPSP) and on the subsequent generation of action potentials (population spike; PS). We observed infraslow fluctuations in low-frequency (0.5–9 Hz) and high-frequency (40–100 Hz) band-limited power (BLP) within the hippocampus that occur anti-phase to observed infraslow fluctuations in the magnitude of fEPSP slope yet occur in-phase with infraslow fluctuations in PS amplitude. Thus, ongoing infraslow SBA appears to contribute to evoked response variability by dynamically altering fEPSP-PS coupling of hippocampal neurons.

## Materials and Methods

### Surgery

Three- to four-month-old, male Sprague Dawley rats (*n* = 17, Harlan) were housed individually under standard laboratory conditions (12/12 h light/dark cycle, access to food and water *ad libitum*). Immediately before surgery, rats were treated with an antibiotic (penicillin; 100,000 units/kg) and NSAID analgesic (flunixin; 2.5 mg/kg). Under isoflurane anesthesia (5% induction, 2.5% maintenance) electrodes were bilaterally implanted as previously described (Korol and Gold, 2008) to record evoked field potentials within the dentate gyrus following electrical stimulation of the medial perforant pathway. Specifically, Teflon-coated stainless steel wires (0.003’’ bare diameter, A-M Systems) were implanted into the hilus of the dentate gyrus (from bregma: anteroposterior (AP), ∓3.5 mm; mediolateral (ML), ±2 mm; dorsoventral (DV), ∼3.5-4 mm) and referenced to screws affixed to the skull to record extracellular field potentials. Two additional Teflon-coated stainless steel wires, with roughly 500 µm of insulation removed at the end, were implanted into the medial perforant pathways (AP, -8.1 mm; ML, ±4.2 mm; DV, ∼3-3.5 mm) and referenced to screws affixed to the skull to serve as stimulating electrodes. Final positions for both stimulating and recording electrodes were adjusted to ensure correct placement by maximizing the magnitude of the evoked potential. Recording, stimulating, and screw electrodes were capped with gold Amphenol pins, placed inside a nine-pin ABS plug (Ginder Scientific), and affixed to the skull with dental acrylic. Postoperative care included topical administration of antibacterial ointment (bacitracin) to the surgical site and access to analgesic for 24 h following surgery (Children’s Ibuprofen; 2.35 ml in 500-ml water bottles). All rats recovered undisturbed for at least 7 d following surgery before any additional experimental procedures were conducted. These methods and those below were conducted in accordance with the National Institutes of Health Guide for the Care and Use of Laboratory Animals and were approved by the Syracuse University Institutional Animal Care and Use Committee.

### Electrophysiological recordings

Evoked field potentials following electrical stimulation of the medial perforant pathway were recorded from each rat in its home cage. All electrode leads were connected to a commutator with flexible wiring to enable unobstructed movement throughout the home cage. The timing of electrical stimulations was controlled by computer program (Signal 4; Cambridge Electronic Design) and an attached microcontroller (Micro1401; Cambridge Electronic Design). Monopolar stimulations (square pulses, 200-µs duration, typically between 0.1 and 1 mA) were produced by battery-operated stimulus isolator units (World Precision Instruments A365) and delivered to the stimulating electrodes. Evoked field potentials and spontaneous electroencephalographic (EEG) activity were amplified (200×) and filtered (high-pass: 0.3 Hz; low-pass: 3 kHz) with ac-amplifiers (Grass Technologies Model P5111K) and the resultant signal recorded by the computer (sampling frequency = 10,000 Hz).

On the first day of recording for each rat, the baseline stimulation intensity was determined. First, a range of stimulations was given to find the minimum stimulation intensity to produce a fEPSP and to find the intensity at which no further increases in fEPSP could be elicited (maximum intensity). An input/output curve of 10 stimulation intensities evenly interspersed between the minimum and maximum intensity was then established by recording three evoked responses at each stimulation intensity (20s interstimulation interval). The slope of the fEPSP was quantified and in all cases increased as a function of increasing input intensity. From this input/output curve, the intensity of baseline stimulation was determined by selecting the stimulation intensity that produced ∼40% of the maximal fEPSP slope to ensure that future recordings would be sensitive to either increases or decreases in fEPSP slope. Baseline recordings (30 stimulations delivered at 0.05 Hz) were performed daily for at least four consecutive days to confirm that stable evoked responses (daily fEPSP means not differing by more than ±5%) could be produced. On the day following establishment of a stable baseline, two 28-min recording sessions (10-15 min between each session) were performed. Herein, electrical stimulations at baseline intensity were delivered throughout at 0.25 Hz (*n* = 10 rats). To test whether results from this population depended on stimulation frequency, all experimental procedures and analyses were performed on a second group of rats (*n* = 7) that only differed in their stimulation frequency (0.10 Hz). Evoked responses and spontaneous EEG activity were recorded. All recordings were taken between 4 and 7 h after vivarium light onset. Additionally, the behavioral state of the rat was monitored and classified as either quiet waking (eyes-open, immobile), active waking (exploratory behavior), behaviorally-defined sleep (eyes-closed, immobile), or grooming.

Of note, rats in the present study were previously part of a separate study investigating mechanisms by which epinephrine modulates long-term potentiation (our unpublished observations). For the present study, the electrophysiological recordings described above occurred: (1) at least 7 d following the completion of any previous experiments, (2) only after the baseline evoked response was stable for four consecutive days, and (3) typically approximaltey four weeks after surgery.

### Experimental design, data processing, and statistical analyses

All data were analyzed off-line with custom scripts in Mathworks MATLAB. Additional statistics were performed with Statistica 6 (Statsoft). Two parameters of the evoked response were quantified: (1) the fEPSP, produced by the initial depolarization of neurons within the dentate gyrus following stimulation; and (2) the PS, produced by the collective firing of action potentials within the population (McNaughton and Barnes, 1977). The fEPSP was quantified by measuring the slope of the large positive deflection following cessation of the stimulation artifact. fEPSP slopes were calculated within a ∼0.4- to 0.5-ms window following slope onset to avoid potential confounding effects of PS onset variability on fEPSP slope. PS amplitude was quantified by first drawing a tangent between to the two apices of the evoked response and then calculating the amplitude of a vertical line drawn between the trough of the PS and this tangent line. To facilitate comparisons across rats, fEPSP slope and PS amplitude were normalized within each rat to their respective mean values across a recording session. fEPSP slope could be quantified for all rats while PS amplitude could be calculated for 15 of 17 rats.

Spontaneous EEG activity was recorded throughout the two, 28-min recording sessions. EEG signals were down sampled to 200 Hz and 60 Hz line noise was filtered with a zero-phase, Chebyshev Type II bandstop filter. Manual rejection of movement artifacts present in the EEG data was performed by visual inspection of EEG data and their corresponding power spectra in 4-s epochs. Epochs with large, atypical voltage deflections and/or prominent power <0.5 Hz (frequencies that cannot be recorded with our ac-amplifiers) were classified as artifact and were not included in any future analyses. Overall, <5% of epochs were rejected. Power spectra were calculated via Welch’s method (hamming window, 0.25 Hz resolution) for each 4-s epoch of spontaneous EEG data. EEG BLP was calculated by averaging the total power present within distinct frequency bands (δ: 0.5–4 Hz; θ: 5–9 Hz; α: 10–18 Hz; β: 22–30 Hz; high-β: 30.25–40 Hz; low-γ: 40.25–59 Hz; mid-γ: 61–80 Hz; high-γ: 80.25–100 Hz). As similar results were obtained throughout this study from neighboring frequency bands, BLP for three broad frequency bands was also calculated (low frequencies: 0.5–9 Hz; middle frequencies: 10–40 Hz; high frequencies: 40.25–100 Hz). Time series of BLP were created across the entire recording session and power spectra of these time series were obtained as above to assess whether infraslow (<0.1 Hz) fluctuations in BLP were present.

Multiple approaches were taken to quantify the relationships between hippocampal evoked response variability and SBA. Two of these approaches were conducted in the time domain. First, we calculated Pearson’s correlation coefficients between BLP at each frequency band in the 4 s preceding each evoked response and either fEPSP slope or PS amplitude for each individual rat. Second, we binned all evoked responses into quartiles as a function of the amount of BLP in the preceding 4 s. The average fEPSP slope and PS amplitude were calculated within each quartile and the significance of these results was assessed with a repeated measures ANOVA.

Additional analyses were conducted in the frequency domain. Missing data points in BLP time series (due to previous artifact rejection) were linearly interpolated to enable digital filtering. Time series of BLP, fEPSP slope, and PS amplitude across each recording session were filtered in the infraslow range with a zero-phase, Type II Chebyshev filter (band pass: 0.004–0.025 Hz). For each signal, we calculated the angle of the complex vector obtained from the Hilbert transform to provide a measure of instantaneous phase (Vanhatalo et al., 2004). Instantaneous phase differences (IPDs) were calculated as follows: IPD = eˆ(i*(P_1_-P_2_)), wherein P_1_ and P_2_ are the instantaneous phase of the two signals being compared. The average of IPD is a complex number in which the imaginary component is the average phase difference and the real part is the phase-locking factor (PLF). The PLF ranges from 0 to 1, with values near 0 representing a uniform phase distribution and values near 1 indicating perfect phase synchrony (Vanhatalo et al., 2004). To assess the significance of the obtained IPD and PLF, distributions of random IPDs and PLFs were created by repeating the same analyses described above 1000 times for each rat with randomized fEPSP and PS data. Overall, actual IPDs and PLFs were compared to the randomized data to determine whether the relationships observed in the actual data reflect true phase locking or rather, arise simply due to chance.

We used a complementary method to address further the relationship between infraslow BLP activity and evoked response magnitude. Herein, evoked responses were binned as a function of the instantaneous phase of infraslow BLP. fEPSP slope and PS amplitude were averaged within each bin. Lastly, the ratio of fEPSP slope and PS amplitude was calculated as a measure of fEPSP-PS coupling. fEPSP-PS coupling reflects the amount of output produced (PS) by a given input (fEPSP; Daoudal and Debanne, 2003; Zsiros and Hestrin, 2005). To investigate how ongoing SBA affects fEPSP-PS coupling, we binned and averaged this fEPSP/PS ratio as a function of infraslow BLP phase.

Throughout this study, all results are expressed as the arithmetic mean ± SEM with the exception of phase differences which are expressed as the circular mean ± circular SD (Berens, 2009). Circular means are presented as positive angles along the unit circle between 0 and 2π.

## Results

### Extensive variability of evoked response and spontaneous EEG activity

**Figure 1.**
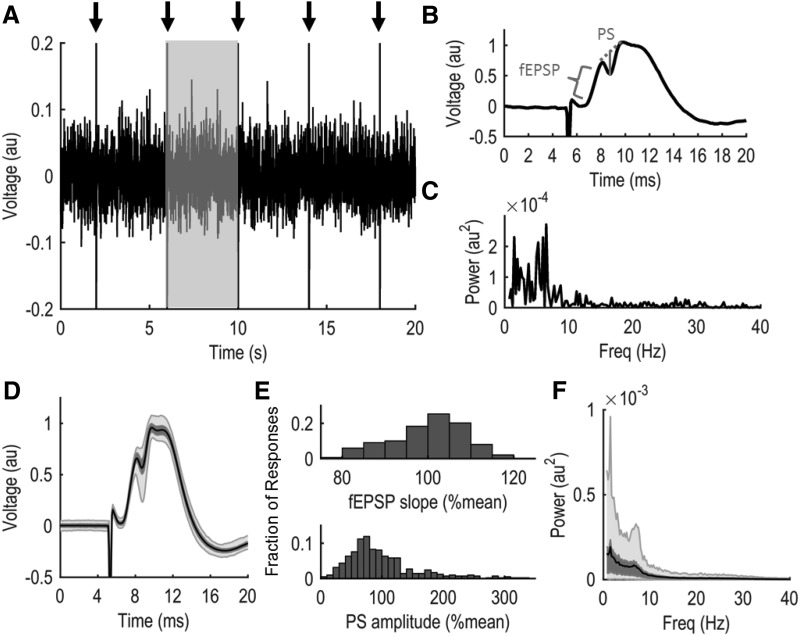
Extensive variability of evoked response magnitude and spontaneous EEG power during a 28-min continuous recording session. ***A***, Twenty seconds of raw EEG data from an individual rat. Arrows denote electrical stimulation to produce an evoked response. ***B***, A typical evoked response. To quantify the evoked response, fEPSP slope is calculated within the bracketed region while PS amplitude (solid gray line) is the amplitude from response trough to the tangent line connecting response peaks (dotted line). ***C***, Power spectrum of spontaneous EEG activity from the 4-s shaded region in ***A***. ***D***, Average evoked response from all 420 stimulations during a recording session in a single rat. ***E***, Extensive variability in the evoked response is observed in both the fEPSP slope (top) and PS amplitude (bottom). ***F***, The power spectrum of SBA averaged across all 4-s epochs of the recording session. Power spectra were calculated from 0.5 to 100 Hz but are only depicted to 40 Hz here for enhanced visualization. In ***D***, ***F***, dark shaded regions encompass the middle 50% of all observed values and light shaded regions encompass 95% of all observed values. Similar variability in both evoked responses magnitude and EEG power was observed when the stimulation frequency was 0.10 Hz instead of 0.25 Hz (see Results).

The slope of the evoked fEPSP and amplitude of PS can be used to quantify the response of a neuronal population to incoming stimulation. Increased stimulation intensity typically results in an increased fEPSP slope and elevated PS amplitude. Notably, evoked response magnitude can vary considerably even when stimulation intensity is held constant. During 28-min recording sessions (for experimental design, see Materials and Methods; Fig. 1), we recorded 420 evoked responses in the dentate gyrus following stimulation of the perforant pathway (0.25 Hz; *n* = 10). Extensive trial-to-trial variability of the fEPSP slope and PS amplitude was observed (Fig. 1), with a large average SD of the fEPSP (11.66 ± 2.10% mean fEPSP) and PS (37.93 ± 5.00% mean PS) across all animals. We also observed extensive evoked response variability when rats were stimulated at 0.10 Hz (*n* = 7), including large average SDs of the fEPSP (14.69 ± 2.37% mean fEPSP) and PS (22.11 ± 3.51% mean PS). To assess whether SBA exhibits similar variability to the evoked responses, we calculated the amount of broadband EEG power (0.5–100 Hz) in 4-s epochs preceding each evoked response. Four-second epochs of broadband EEG power also exhibited substantial variability (Fig. 1; average SD: 41.43 ± 3.56% mean total power) raising the possibility that changes in SBA are associated with the observed evoked response variability.

fEPSP slope and PS amplitude variability do not appear to arise primarily as consequences of systemic changes in perforant path – dentate gyrus connectivity resulting from prolonged stimulation. While fEPSP slopes and PS amplitudes recorded from rats stimulated at 0.25 Hz were on average smaller in the second half of recordings (97.35 ± 1.25%, *t*_(19)_ = -2.13, *p* = 0.047 and 87.45 ± 3.53%, *t*_(17)_ = -3.55, *p* = 0.0025, respectively) than in the first half of recordings, linear regression reveals that time of stimulation only account for 3.3% of the total variance in fEPSP slope and 13.2% of total PS amplitude variance. Moreover, first and second half fEPSP slopes were not significantly different (*t*_(13)_ = -0.68, *p* = 0.51) in 0.10 Hz stimulated rats with time of stimulation only accounting for 1.1% of total fEPSP variance in this population. Likewise, PS amplitudes in 0.10 Hz stimulated rats were not significantly affected by time of stimulation (*t*_(11)_ = -0.79, *p* = 0.44, with time of stimulation accounting for only 2.1% of total PS amplitude variation).

### Antecedent EEG activity is associated with evoked response variability

**Figure 2.**
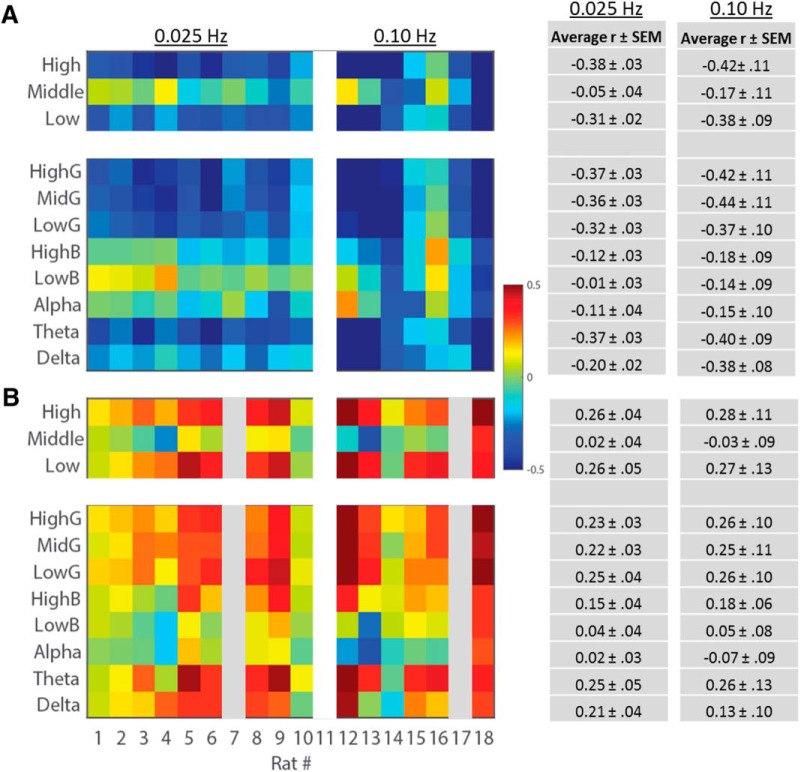
Correlations between antecedent EEG power and fEPSP slope (***A***) or PS amplitude (***B***) at stimulation frequencies of either 0.25 or 0.10 Hz. Heat maps depict correlations for each animal for each frequency bin while reported r values reflect the average r value observed across all rats. Low- and high-frequency BLP and fEPSP slope are negatively correlated while these same frequency bins are positively correlated with PS amplitude. PS amplitude could not be consistently quantified for rat 7 or 17. For frequency bin labels, G = γ; B = β.

We calculated the correlations between BLP in the 4 s preceding each evoked response and the ensuing fEPSP or PS. Similar patterns of correlation were observed across all rats regardless of stimulation frequency wherein the strength of correlation varied as a consequence of BLP frequency and the component of the evoked response (Fig. 2). Specifically, negative correlations were typically observed between the fEPSP slope and both low-frequency BLP (0.5–9 Hz; average correlation: *r*_0.25_ = -0.31 ± 0.02, *r*_0.10_ = -0.38 ± 0.09) and high-frequency BLP (40.25–100 Hz; *r*_0.25_ = -0.38 ± 0.03, *r*_0.10_ = -0.42 ± 011). By contrast, correlations between fEPSP slope and middle-frequency BLP (10–40 Hz) were heterogeneous across individual rats and on average small (*r*_0.25_ = -0.05 ± 0.04, *r*_0.10_ = -0.17 ± 0.11) . A similar frequency dependence was observed between BLP and the PS amplitude. PS amplitude, however, was positively correlated with preceding low-frequency BLP (*r*_0.25_ = 0.26 ± 0.04, *r*_0.10_ = 0.27 ± 0.13) and high-frequency BLP (*r*_0.25_ = 0.26 ± 0.05, *r*_0.10_ = 0.28 ± 0.11). Again, these correlations were not evident when comparing PS amplitude and middle-frequency BLP (*r*_0.25_ = 0.02 ± 0.04, *r*_0.10_ = -0.03 ± 0.09). Thus, preceding SBA in the low and high frequencies was negatively correlated with fEPSP slope and positively correlated with PS amplitude.

**Figure 3.**
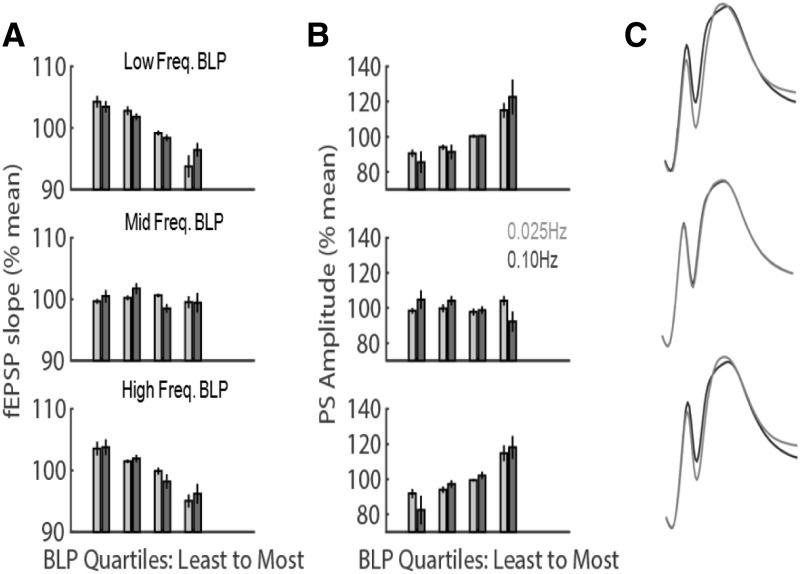
fEPSP slope (***A***) and PS amplitude (***B***) as a function of preceding low-, middle-, or high-frequency BLP quartiles and stimulation frequency (0.25 or 0.10 Hz). ***C***, Average evoked responses from an individual rat for responses following spontaneous EEG epochs that contained the least (black) and most (gray) BLP for each condition.

To quantify the relationships between BLP and evoked response magnitude further, all 4-s epochs of spontaneous activity were subdivided into BLP quartiles and the average fEPSP slope or PS amplitude was calculated within each quartile (Fig. 3). Consistent with the correlations above, fEPSP slope and PS amplitude varied in association with amount of BLP in the low and high, but not middle, frequencies. Evoked responses following epochs that contained the least low-frequency power had larger fEPSP slopes and smaller PS amplitude than responses following epochs that contained the most power (for example evoked responses, see Fig. 3). A similar trend was observed across high-frequency BLP quartiles, but was absent across middle-frequency BLP quartiles. Overall, when characterizing fEPSP variability, a significant main effect of BLP quartile (*F*_(3,45)_ =16.94, *p* = 1.66 × 10^−7^) and a significant interaction between BLP quartile and BLP frequency (*F*_(6,90)_ = 7.61, *p* = 1.28 × 10^−6^) were observed. These effects appear to be independent of stimulation frequency, as we did not observe significant interactions between stimulation frequency and either BLP quartile or the three-way interaction of quartile, BLP frequency, and stimulation frequency (*F*_(3,45)_ = 0.84, *p* = 0.48, and *F*_(6,90)_ = 0.81, *p* = 0.56). When characterizing PS variability, a significant main effect of BLP quartile (*F*_(3,39)_ = 12.56, *p* = 6.86 × 10^−6^) and a significant interaction between BLP quartile and BLP frequency (*F*_(6,78)_ = 5.65, *p* = 6.51 × 10^−5^) was also observed. Again, stimulation frequency did not appear to alter these effects (interaction, stimulation frequency by BLP quartile: *F*_(3,39)_ = 0.20, *p* = 0.90; interaction, stimulation frequency by BLP quartile by BLP frequency: *F*_(6,78)_ = 1.21, *p* = 0.31)

### Infraslow fluctuations in EEG activity are associated with evoked response variability

**Figure 4.**
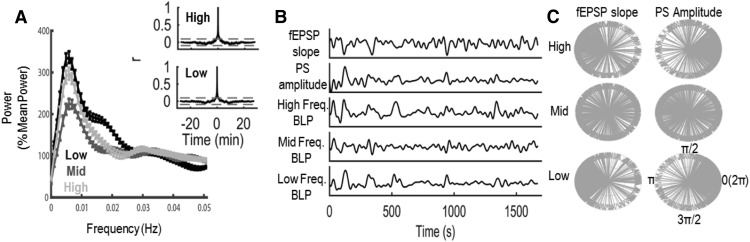
Phase-locking of EEG BLP, fEPSP slope, and PS amplitudes. ***A***, Average power spectra (*n* = 17 rats) of low-, middle-, and high-frequency BLP during the entire recording session. Insets, Autocorrelograms for infraslow-filtered high- and low-frequency BLP. Gray dotted lines depict 95% confidence intervals. ***B***, Spontaneous fluctuations in evoked response magnitude and EEG BLP are evident in infraslow filtered time series data from a single rat. ***C***, IPDs calculated at each data point across the time series data depicted in ***B***. Each gray arrow represents the phase difference calculated at a single time point. fEPSP and low-/high-frequency BLP are typically out of phase (π radians), while PS amplitude and low-/high-frequency BLP are typically in phase [0(2 π) radians]. No clear phase locking is present between middle-frequency BLP and fEPSP slope or PS amplitude.

The above analyses solely focus on the relationships between evoked responses and the preceding 4-s epochs of EEG BLP and as such are unable to determine the source(s) of the variability of BLP and/or the evoked responses. Previous reports indicate that neocortical BLP exhibits infraslow (< 0.1 Hz) fluctuations in total power that are associated with changes in neuronal excitability (Vanhatalo et al., 2004). To investigate whether the observed variability in BLP and evoked response were, in part, driven by fluctuations on a similar timescale, we created time-series of EEG BLP, fEPSP slope, and PS amplitude across each recording session. Figure 4 depicts the power spectra of the time-series of EEG BLP for low, middle, and high frequencies. Across infraslow frequencies (0.005–0.02 Hz), we observed broad peaks in the power spectra for low- and high-frequency BLP that were absent in the BLP of middle frequencies. Consequently, we filtered the EEG BLP time series in the infraslow range. Consistent with the broad infraslow peaks present in the power spectra, autocorrelograms of infraslow-filtered low- and high-frequency BLP (Fig. 4), which lack discrete peaks of high correlation except those surrounding zero lag, further demonstrate that this infraslow activity is best characterized as a nonharmonic fluctuation rather than a continuous harmonic oscillation. Specifically, cycle-to-cycle variation in period length occurred throughout our recording sessions, with average period lengths of 1.56 ± 0.07 and 1.37 ± 0.07 min, for infraslow-filtered low- and high-frequency BLP, respectively. Additionally, while these nonharmonic fluctuations were continuously present throughout all recording periods, the peak-to-trough amplitude of each cycle also varied considerably: The average standard deviation of within-session peak-to-trough amplitudes for high-frequency BLP was 38.79 ± 12.87% and for low-frequency BLP was 60.50 ± 21.42% of mean peak-to-trough amplitude. Thus, it appears as though very slow, nonharmonic fluctuations in SBA are present in low- and high-frequency BLP and, moreover, that the period length and amplitude of these fluctuations can vary across time.

**Figure 5.**
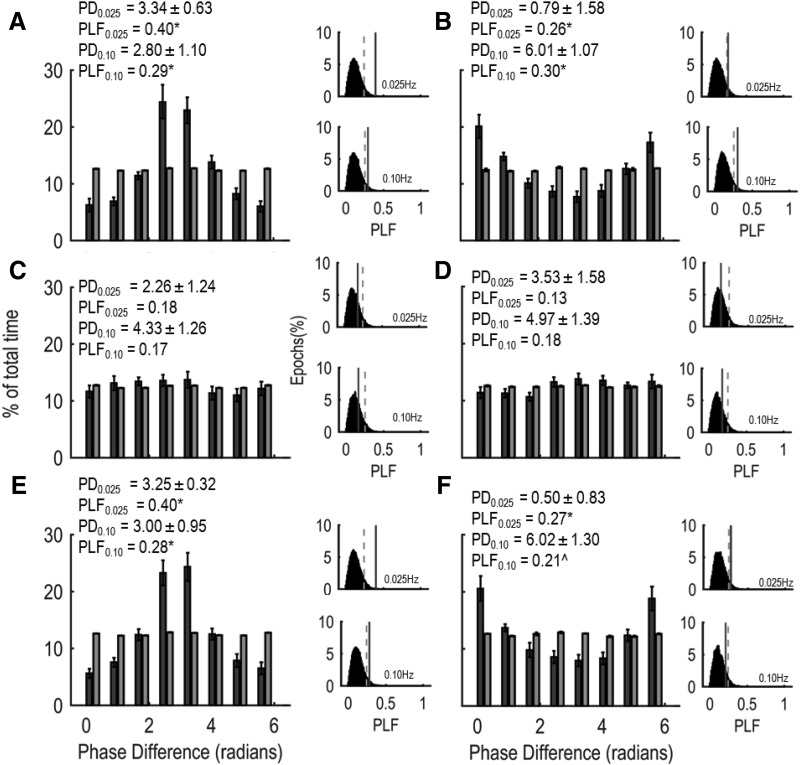
IPD between low-frequency (***A***, ***B***), middle-frequency (***C***, ***D***), and high-frequency (***E***, ***F***) BLP and fEPSP or PS for rats stimulated at 0.25 Hz (similar phase distributions were observed for 0.10 Hz stimulated rats). Dark bars depict the distribution of actual observed phase differences while light bars depict the distribution of phase differences observed between BLP and shuffled fEPSP or PS data. fEPSP slopes are predominantly antiphase with high/low BLP, while PS amplitudes are predominantly in phase with high/low BLP. Insets depict the distribution of PLFs calculated between BLP and shuffled fEPSP or PS data (histogram), the 0.05 critical value obtained from these distributions (dotted line), and the PLF calculated from observed data (solid gray line). PD = circular mean phase difference ± circular SD. Phase locking: *p < 0.05, ^*p* < 0.10.

Infraslow EEG BLP may be associated with the observed fEPSP slope and PS amplitude variability. To assess this possibility, we filtered fEPSP slope and PS amplitude time series in the infraslow range and calculated IPDs between each of these measures (for example data from an individual rat, see Fig. 4). To quantify these phase differences across all rats, we calculated the distribution of phase differences between BLP and either fEPSP slope or PS amplitude for our observed data and compared these distributions to random distributions obtained by comparing phase differences of our shuffled data. As seen in Figure 5, infraslow fluctuations in fEPSP slope were typically antiphase to infraslow fluctuations in low-frequency BLP [circular phase difference mean (PD) in radians ± circular SD: PD_0.25_ = 3.34 ± 0.63, PD_0.10_ = 2.80 ± 1.10] and high-frequency BLP (PD_0.25_ = 3.25 ± 0.32, PD_0.10_ = 3.00 ± 0.95). To assess the significance of these phase differences, we calculated the PLF for the observed data and compared these values to PLFs calculated from shuffled data. Significant phase-locking (i.e., experimentally observed PLFs >95% of all PLFs obtained from 1000 iterations of shuffled data) between fEPSP slope and both low-frequency BLP (PLF_0.25_ = 0.40; *p* < 0.01, PLF_0.10_ = 0.29; *p* < 0.05) and high-frequency BLP (PLF_0.25_ = 0.40; *p* < 0.01, PLF_0.10_ = 0.28; *p* < 0.05) was observed. In contrast, significant phase locking was absent between infraslow fluctuations of fEPSP slope and middle-frequency BLP (PLF_0.25_ = 0.18; *p* > 0.05, PLF_0.10_ = 0.13; *p* > 0.05). The relationship between PS amplitude and spontaneous BLP exhibited a similar frequency dependency to the relationship between fEPSP slope and BLP (Fig. 5). However, infraslow fluctuations in PS amplitude and low- and high-frequency BLP were in phase (PD_0.25_: 0.79 ± 1.58; 0.50 ± 0.83, respectively, PD_0.10_: 6.01 ± 1.07; 6.02 ± 1.30, respectively). Again, this phase locking was significant, or trended significant, for low-frequency (PLF_0.25_ = 0.26, *p* < 0.05, PLF_0.10_ = 0.30 *p* < 0.05) and high-frequency (PLF_0.25_ = 0.27, *p* < 0.05, PLF_0.10_ = 0.21, 0.05 < *p* < 0.10) BLP, but not statistically significant for middle-frequency BLP (PLF_0.25_ = 0.13, *p* > 0.05, PLF_0.10_ = 0.18, *p* > 0.05).

**Figure 6.**
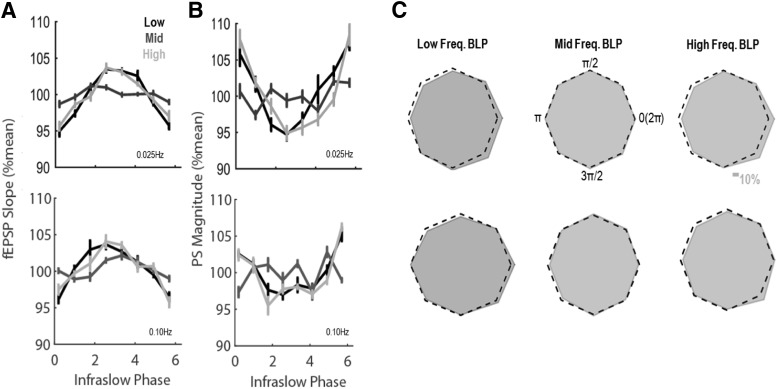
fEPSP slope (***A***) and PS amplitude (***B***) vary as a function of the phase of infraslow fluctuations in low- and high-frequency BLP but are not significantly altered by infraslow changes in middle-frequency BLP. ***C***, The opposing effects of infraslow BLP phase on fEPSP slope and PS amplitude produce variations in EPSP-PS coupling across infraslow cycles in rats stimulated at either 0.25 Hz (top row) or 0.10 Hz (bottom row). Dotted lines depict unitary EPSP-PS coupling at all phases, and gray regions depict observed coupling (regions that exceed dotted lines indicate stronger EPSP-PS coupling than average, while regions that do not reach dotted lines indicate weaker EPSP-PS coupling). Scale bar represents a change of 10% of the mean EPSP-PS coupling.

To quantify the effects of the ongoing phase of infraslow brain activity on evoked response magnitude, fEPSP slope and PS amplitude for each evoked response were binned and averaged as a function of ongoing infraslow BLP phase (Fig. 6). A significant main effect of infraslow phase (*F*_(7105)_ = 16.87, *p* = 9.10 × 10^−15^) and a significant interaction between phase and BLP frequency (*F*_(14,210)_ = 4.08, *p* = 2.90 × 10^−6^) was found to affect fEPSP slope. No significant interactions between stimulation frequency and infraslow phase (*F*_(7105)_ = 0.32, *p* = 0.95) or between stimulation frequency, infraslow phase, and BLP frequency (*F*_(14,210)_ = 1.12, *p* = 0.34) were observed to affect fEPSP slope. Infraslow phase significantly affected PS amplitude (*F*_(7,91)_ = 4.39, *p* = 3.09 × 10^−4^) and a significant interaction between phase and BLP frequency (*F*_(14,182)_ = 2.42, *p* = 0.004) was found to affect PS amplitude. No significant interactions between stimulation frequency and infraslow phase (*F*_(7,91)_ = 1.56, *p* = 0.15) or between stimulation frequency, infraslow phase, and BLP frequency (*F*_(14,182)_ = 0.94, *p* = 0.52) were observed to affect PS amplitude. Overall fEPSP slope was steepest and PS amplitude the smallest during nadirs of the infraslow fluctuation in either low- or high-frequency BLP. The instantaneous phase of middle-frequency BLP did not appear to affect either the fEPSP slope or PS amplitude. Thus, spontaneous infraslow fluctuations in low- and high-frequency BLP are associated with evoked response variability and appear independent of stimulation frequency.

The opposing effects of infraslow BLP fluctuations on the fEPSP and PS indicate that coupling of incoming depolarization (fEPSP) with output (PS) varies dynamically in the dentate gyrus as a function of ongoing SBA. As evident in Figure 6, EPSP-PS coupling changed in association with changing phase of infraslow fluctuations in low-/high-frequency BLP but showed no association with fluctuating BLP in the middle frequencies. Specifically, the phase of infraslow BLP significantly affected the degree of EPSP-PS coupling (*F*_(7,91)_ = 13.96, *p* = 3.62 × 10^−12^) and moreover, the effect of phase was modulated by the frequency of BLP (*F*_(14,182)_ = 6.95, *p* = 2.38 × 10^−11^). No significant effects of stimulation frequency were observed (interaction between stimulation frequency and infraslow phase: *F*_(7,91)_ = 0.49, *p* = 0.84, interaction between stimulation frequency, infraslow phase, and BLP quartile: *F*_(14,182)_ = 0.81, *p* = 0.66). Thus, independent of stimulation frequency, EPSP-PS coupling is maximal at the peak of infraslow fluctuations in low- or high-frequency BLP (∼10-15% higher than average) and minimal during the nadir of infraslow fluctuations in low- or high-frequency BLP (∼10-15% lower than average).

### Changes in behavioral state do not appear responsible for infraslow EEG fluctuations and associated evoked response variability

Hippocampal evoked response magnitude varies in association with behavioral state (Coenen, 1975; Winson and Abzug, 1977; Green et al., 1990). To determine whether infraslow variability observed in the present study is a reflection of behavioral state fluctuations, we assessed the relationships between behavior, evoked response magnitude, and infraslow activity. Behavioral data were only recorded for rats stimulated at 0.25 Hz and thus all behavioral data presented are from this group only. Throughout the recording sessions, rats exhibited quiet wakefulness for the majority of time observed (66.33 ± 10.08%) with the remainder of time filled with exploratory waking (23.00 ± 9.21%), behaviorally-defined sleep (8.00 ± 8.99%) and active grooming (2.67 ± 3.67%). Similar to previous reports (Green et al., 1990; Moser et al., 1993), fEPSP slopes evoked during periods of active waking were significantly steeper than those evoked during quiet waking (active fEPSP slope 107.87 ± 2.92% of quiet waking; *t*_(9)_ = 2.70, *p* = 0.024), while PS amplitudes trended lower (active PS amplitude 79.33 ± 8.77% of quiet waking; *t*_(8)_ = 2.04, *p* = 0.076).

This state-dependent variability, however, does not appear well-suited to explain infraslow variability in evoked response magnitude discussed above. Infraslow fluctuations in evoked response magnitude were observed across all rats, yet rats varied considerably in the amount of time spent in each behavioral state (e.g., time spent in quiet waking ranged from 43.33% to 96.67% of total recording time). Moreover, the average bout duration for quiet waking (11.96 ± 2.71 min) and active waking (4.47 ± 0.82 min) were both considerably longer than the average period of infraslow fluctuations in fEPSP slope (1.37 ± 0.04 min) or PS amplitude (1.27 ± 0.03 min). Multiple infraslow cycles, therefore, can occur without alternation of behavioral state.

To assess this possibility directly and to determine whether the infraslow oscillations in fEPSP slope or PS amplitude described above were related to behavioral state dependent changes in evoked response magnitude, we examined whether the distribution of instantaneous infraslow phases deviated from a uniform distribution during periods of quiet or active waking. If behavioral state were biasing the infraslow phase distribution, we would expect to see a nonuniform phase distribution within each behavioral state. Conversely, a uniform distribution indicates that complete cycles of infraslow oscillations readily occur without changes in behavioral state and therefore it is unlikely that the infraslow oscillations are driven by behavioral state. For both fEPSP slope and PS magnitude, we did not observe significant deviations from a uniform distribution in either active waking (Rayleigh_z_ = 1.42, *p* = 0.243) or quiet waking (Ralyeigh_z_ = 1.73, *p* = 0.178). Thus, we did not find evidence to support that the infraslow fluctuations in evoked response magnitude described herein are byproducts of behavioral state changes. Moreover, behavioral state did not appear to affect infraslow fluctuations of low- or high-frequency BLP as average infraslow cycle durations (low: *t*_(9)_ = 1.15, *p* = 0.28; high: *t*_(9)_ = 0.44, *p* = 0.67) and mean peak-to-trough amplitudes (low: *t*_(9)_ = 0.59, *p* = 0.57; high: *t*_(9)_ = 0.71, *p* = 0.50) did not significantly differ between active and quiet waking.

## Discussion

We observed extensive variability of electrically evoked responses within the hippocampus that were independent of stimulation frequency (0.1 or 0.25 Hz). The responses were associated with antecedent levels of both low- and high-frequency BLP. Throughout our recording periods, low- and high-frequency BLP exhibited spontaneous infraslow fluctuations (<0.1 Hz) that contributed to the observed trial-to-trial variability of the evoked response by dynamically altering the generation of action potentials in response to postsynaptic depolarization (fEPSP-PS coupling). Together, these results provide evidence that (1) the hippocampus exhibits infraslow fluctuations in SBA with a periodicity similar to those previously described in neocortex, and (2) the effects of infraslow SBA on the processing of incoming stimuli may arise as a consequence of changes in fEPSP-PS coupling.

Infraslow fluctuations in spontaneous BOLD fMRI signals (Smith et al., 2009; Raichle, 2015), full band EEG (Vanhatalo et al., 2004), and BLP of higher frequencies (>0.1 Hz; Monto et al., 2008) have been reported throughout neocortex. Our observation of slow (0.004–0.025 Hz), nonharmonic oscillatory activity with extensive variation in cycle-to-cycle peak-to-trough amplitudes provides novel empirical support establishing the presence of similar spontaneous infraslow activity within hippocampal networks. Notably however, this hippocampal infraslow activity is associated with a distinct pattern of modulation of BLP of higher frequencies with fluctuations in low (0.5–9 Hz) and high (40.25–100 Hz) frequency BLP that were largely absent in middle-frequency BLP (10–40 Hz).

The coordinated activity of low- and high-frequency hippocampal activity may, in part, arise from extensive θ-γ coupling characteristic of hippocampal ensembles (Jensen and Colgin, 2007; Shirvalkar et al., 2010). Prominent θ-activity within the hippocampus occurs as a consequence of its distinct cellular composition (replete with a diversity of morphologically and functionally distinct inhibitory interneurons; (Freund and Buzsáki, 1996), and connectivity (e.g., medial septal cholinergic input; Mitchell et al., 1982). Coordination of rhythmic pyramidal cell excitation across the θ-cycle with inhibitory signaling couples ongoing θ-activity to the generation of γ-oscillations (Wulff et al., 2009; Colgin, 2015). These intrinsic hippocampal characteristics may therefore account for the frequency-dependent associations of our observed infraslow SBA; this observation is consistent with the region-dependent coupling of high-frequency BLP and infraslow activity first identified within functionally distinct resting state fMRI networks (Mantini et al., 2007). Infraslow SBA, therefore, is present across many brain regions although its effects on the power of higher frequency oscillatory activities appear to be constrained by region-specific functional connectivity and/or cellular phenotype.

Despite the ubiquity of infraslow activity throughout the brain, the physiologic mechanisms responsible for infraslow SBA are not well characterized. Regionally heterogeneous infraslow activity could result from (1) stochastic resonance leading to the formation of dynamic assemblies and/or (2) intrinsic oscillatory activity present within neuronal or astrocytic cells at infraslow timescales (Deco and Corbetta, 2011; Palva and Palva, 2012; Zylbertal et al., 2017). Biochemically, infraslow activity has been associated with activation of adenosine A1 receptors (Ĺórincz et al., 2009; Lindquist and Shuttleworth, 2012), regenerative calcium waves within networks of astrocytes (Kuga et al., 2011), and activity of subcortical neuromodulatory centers including the locus coeruleus and raphe nucleus (Filippov et al., 2004). These physiologic signals may ultimately modulate neuronal excitability across infraslow timescales through the emergence of spontaneous fluctuations in resting membrane potential (Chan et al., 2015).

The observed changes in fEPSP-PS coupling that were contingent on the phase of infraslow SBA could arise as a direct consequence of these membrane potential fluctuations. fEPSP-PS coupling has previously been shown to vary along with intrinsic changes in postsynaptic excitability (Daoudal and Debanne, 2003; Zsiros and Hestrin, 2005), and moreover is regulated by many of the same neuromodulatory systems detailed above. Specifically, adenosine (Ferguson and Stone, 2010), norepinephrine (Kitchigina et al., 1997), and serotonin (Klancnik and Phillips, 1991) have all been shown to alter fEPSP-PS coupling within the dentate gyrus. Moreover, changes in granule cell excitability have been previously proposed (Bramham, 1998) to explain a similar decoupling of fEPSP and PS amplitude to that observed in the present study. During spontaneous dentate spikes, transient hippocampal phenomena that are associated with the depolarization of granule cells (Penttonen et al., 1997), Bramham (1998) observed decreases in fEPSP slope that were nevertheless associated with increased PS amplitude. Thus, when granule cells within the dentate gyrus are relatively hyperpolarized, electrostatic driving forces, potentially augmented by facilitating effects of enhanced ephaptic coupling (Berretta et al., 2000), may produce enhanced fEPSPs that nevertheless fail to reach the threshold for action potential generation. By contrast, even minimal additional postsynaptic depolarization in a depolarized neuron may generate spiking activity.

Changes in fEPSP-PS coupling of granular neurons could produce widespread alterations in hippocampal spiking activity and consequent information processing. Dentate granule cell firing (i.e., PS) filters entorhinal inputs (i.e., EPSPs), effectively selecting what information can be processed within hippocampal circuitry (Hsu, 2007). The dendritic morphology of granule cells is well-suited to yield a narrow time window during which temporal summation of EPSPs may generate an action potential (Schmidt-Hieber et al., 2007). Precise control of fEPSP-PS coupling is critical for subsequent mossy fiber-CA3 signaling, with high rates of granule cell discharge required to reliably activate CA3 pyramidal neurons (Henze et al., 2002). Ultimately, the precise control of the level and timing of granule cell output contributes to hippocampal function including pattern separation (Leutgeb et al., 2007), spatial navigation (Jung and McNaughton, 1993), and memory formation (Lisman, 1999). The observed changes in fEPSP-PS coupling across infraslow timescales in the present study, therefore, could function to modulate information transfer from entorhinal inputs to hippocampal circuitry. Specifically, infraslow phases associated with weak fEPSP-PS coupling would enable granule cells to act as a stringent, yet highly specific, filter, with only large coincident entorhinal inputs sufficient to generate the high rates of granule firing required to reliably activate downstream CA3 targets (Henze et al., 2002).

Dynamic fEPSP-PS coupling across infraslow scales could likewise underlie previous observations of infraslow SBA modulating information processing and sensory selection (Fontanini and Katz, 2008; Schroeder and Lakatos, 2009; Sadaghiani and Kleinschmidt, 2013). Variations in the neuronal input/output function could account for prominent variability in stimulus detection across infraslow timescales (Monto et al., 2008), and is consistent with previous observations in which SBA led to the formation of distinct neuronal ensembles that incorporate information about both endogenous and exogenous activity (Jones et al., 2007). Endogenous infraslow activity may be subject to modulation itself, thereby providing an active mechanism to tune the response of the brain to incoming stimuli. For example, the amount of resting-state competition at infraslow timescales between task-positive and task-negative brain networks is correlated with individual performance on the Eriksen flanker task (Kelly et al., 2008). Infraslow activity at rest can additionally be modulated by attention (Broyd et al., 2011) or previous experience (Lewis et al., 2009) to further alter information processing within distinct networks. Thus, infraslow SBA, and by extension here, fEPSP-PS coupling, may be subject to both top-down and bottom-up modulation and consequently are well-suited to dynamically alter the brain’s response to incoming stimuli. Notably, in both the present study and in previous characterizations of infraslow activity (for review, see Palva and Palva, 2012), the cycle duration and amplitude of each infraslow fluctuation appears to be highly variable. Such variability may reflect the dynamic regulation of infraslow activity and by extension, the dynamic modulation of fEPSP-PS coupling and information processing. With its well-characterized morphology, circuitry, evoked responses, and our present characterization of infraslow-mediated alterations in fEPSP-PS coupling, the dentate gyrus presents an ideal milieu for further exploration of the dynamic modulation of infraslow activity and resultant effects on brain function.

Behavioral state and higher frequency EEG activity have previously been reported to affect trial-to-trial variability of dentate responses. Dentate evoked responses during slow-wave sleep exhibit smaller EPSPs along with larger and earlier PS’s as compared to inactive waking (Winson and Abzug, 1977; Bramham and Srebro, 1989). Evoked responses are further subject to modulation within the same behavioral state. During periods of active exploration, hippocampal evoked responses recorded from rats have larger EPSPs and decreased PS area (Green et al., 1990). Such changes may be subject to modulation by the environment being explored as evoked responses recorded after rats find a novel object on the hole-board maze have larger PS amplitudes than those recorded from following exploration of empty holes (Kitchigina et al., 1997). Active exploration is typically associated with increased hippocampal θ-power (Coenen, 1975; Green et al., 1990), which may mediate some of the evoked response variability. For example, EPSP slope is larger during periods in which the EEG from anesthetized rats was dominated by the slow-oscillation (0.5–1.5 Hz) as compared to those dominated by θ (3–4 Hz). Moreover, as in the present study, the phase of ongoing oscillatory activity has been associated with evoked response variability; stimulations delivered on the falling phase of either the slow or θ-oscillation produced significantly larger EPSPs than those delivered during the rising phase (Schall et al., 2008). Strikingly, such variability may have important consequences for hippocampal function. High-frequency stimulation delivered at the peak of θ-oscillations resulted in long-term potentiation while similar stimulation delivered at the trough resulted in long-term depression (Hyman et al., 2003). With similar effects on evoked response magnitude, infraslow SBA observed in the present study may complement the mechanisms described above and likewise modulate hippocampal function. Infraslow modulation, however, appears to be a distinct phenomenon that is not directly related to behavioral state-dependent modulations (see Results).

It has been noted that SBA is responsible for the majority of the brain’s energetic demands (Raichle, 2011). The relative abundance of intrinsic activity aligns well with anatomically connectivity wherein the vast majority of synapses do not receive direct sensory input. This predominance of endogenous brain activity exemplifies its important role in regulating brain function. Our observation that spontaneous, infraslow fluctuations are associated with alterations in fEPSP-PS coupling provides a putative mechanism by which sensory selection and information processing can be regulated by ongoing SBA. By modulating these critical functions, infraslow SBA can contribute to behavioral and cognitive flexibility, and, with its potential to coordinate brain activity across diverse temporal and spatial scales, may even contribute to the emergence of consciousness itself (He and Raichle, 2009).

## Synthesis

Reviewing Editor: Satu Palva, University of Helsinki Decisions are customarily a result of the Reviewing Editor and the peer reviewers coming together and discussing their recommendations until a consensus is reached. When revisions are invited, a fact-based synthesis statement explaining their decision and outlining what is needed to prepare a revision will be listed below. The following reviewer(s) agreed to reveal their identity: Clive Bramham, Wickliffe Abraham.

The reviewers found your manuscript revised well according to earlier comments.
